# Orchestrating the Impact of KIR/HLA Interactions on Kidney Transplant

**DOI:** 10.3390/ijms25158228

**Published:** 2024-07-28

**Authors:** Luminița-Ioana Iancu Loga, Ramona Suharoschi, Florin Ioan Elec, Alin Dan Chiorean, Alina Daciana Elec, Adriana Milena Muntean, Mihai Domnuțiu Suciu, Oana Antal, Andreea Teodora Toth, Roxana Liana Lucaciu, Adriana Corina Hangan, Tudor Drugan, Horea Vladi Matei, Lucia Dican

**Affiliations:** 1 Department of Cellular and Molecular Biology, Faculty of Medicine, “Iuliu-Hațieganu” University of Medicine and Pharmacy, 400012 Cluj-Napoca, Romania; luminita.loga@icutr.ro (L.-I.I.L.); chiorean.alin@umfcluj.ro (A.D.C.); hmatei@umfcluj.ro (H.V.M.); 2 Clinical Institute of Urology and Renal Transplantation, 400000 Cluj-Napoca, Romania; alina.elec@icutr.ro (A.D.E.); adriana.muntean@icutr.ro (A.M.M.); mihai.suciu@icutr.ro (M.D.S.); antal.oanna@elearn.umfcluj.ro (O.A.); toth_andreea_teodora@elearn.umfcluj.ro (A.T.T.); lucia.dican@umfcluj.ro (L.D.); 3 Faculty of Food Science and Technology, University of Agricultural Sciences and Veterinary Medicine of Cluj-Napoca, 400372 Cluj-Napoca, Romania; 4 Department of Urology, Faculty of Medicine, “Iuliu-Hațieganu” University of Medicine and Pharmacy, 400012 Cluj-Napoca, Romania; 5 Department of Anesthesiology, “Iuliu-Hațieganu” University of Medicine and Pharmacy, 400012 Cluj-Napoca, Romania; 6 Department of Pharmaceutical Biochemistry and Clinical Laboratory, Faculty of Pharmacy, “Iuliu-Hațieganu” University of Medicine and Pharmacy, 400012 Cluj-Napoca, Romania; liana.lucaciu@umfcluj.ro; 7 Department of Inorganic Chemistry, Faculty of Pharmacy, “Iuliu-Hațieganu” University of Medicine and Pharmacy, 400012 Cluj-Napoca, Romania; adriana.hangan@umfcluj.ro; 8 Department of Medical Informatics and Biostatistics, Faculty of Medicine,“Iuliu-Hațieganu” University of Medicine and Pharmacy, 400012 Cluj-Napoca, Romania; 9 Department of Medical Biochemistry, Faculty of Medicine, “Iuliu-Hațieganu” University of Medicine and Pharmacy, 400012 Cluj-Napoca, Romania

**Keywords:** killer-cell immunoglobulin-like receptors (KIR), human leucocyte antigen (HLA), chronic rejection, kidney transplantation

## Abstract

This study examines the interplay between human leukocyte antigen (HLA) compatibility and killer-cell immunoglobulin-like receptor (KIR) genotypes in influencing kidney transplantation outcomes. Understanding these interactions is crucial for improving graft survival and minimizing rejection risks. We evaluated 84 kidney transplant recipients, dividing them into two groups based on post-transplant outcomes: there were 68 with stable graft function (SGF) and 16 who experienced chronic rejection (CR). Patients were selected based on specific inclusion criteria. HLA mismatches (Class I: HLA-A, -B; Class II: HLA-DR) and KIR genotypes were determined using standard genotyping techniques. Statistical analyses, including logistic regression, were performed to correlate these factors with transplant outcomes. Significant age differences were observed, with younger patients more likely to experience graft rejection, while no significant gender-based differences were noted. A significant correlation was found between Class II mismatches and increased rejection rates, highlighting the importance of HLA-DR compatibility. Further analysis revealed that certain inhibitory KIRs, such as KIR3DL1, were associated with favorable outcomes, suggesting a protective role against graft rejection. These findings were corroborated by evaluating serum creatinine levels over multiple years, serving as a biomarker for renal function post transplant. This study underscores the critical need for meticulous HLA matching and the consideration of KIR genotypes in pre-transplant evaluations to enhance graft survival and minimize rejection risks. Integrating these genetic factors into routine clinical assessments could significantly improve personalized transplant medicine strategies, ultimately enhancing patient outcomes. Further research is needed to explore the underlying mechanisms and validate these findings in larger, diverse populations.

## 1. Introduction

Kidney transplantation has significantly improved outcomes for patients with end-stage renal disease. However, immune-mediated graft rejection remains a significant hurdle, affecting both graft survival and function [1,2,3]. The immune responses involved are influenced by numerous factors, including the compatibility between the recipient’s immune system and the donated organ [4,5]. Particularly, the interactions between killer immunoglobulin-like receptors (KIRs) and human leukocyte antigens (HLAs) play a pivotal role in the recipient’s immune response to the transplanted kidney. These interactions can lead to either the activation or inhibition of natural killer (NK) cells, which are crucial for innate immune surveillance and also affect adaptive immune responses [6,7].

NK cells are critical components of the innate immune system, acting as the first line of defense and serving as a crucial bridge to adaptive immunity. They are not only pivotal in combating infections and tumor cells, but also play ambivalent roles in transplant outcomes, potentially promoting graft damage or contributing to graft tolerance. The functionality of NK cells is heavily modulated by KIRs, which are glycoproteins expressed on the surface of NK cells [8]. These receptors recognize specific motifs on HLA Class I molecules, dictating the activation or inhibition of NK cells. KIRs are categorized based on their structure and function—those with long cytoplasmic tails transmit inhibitory signals, helping to maintain tolerance to self-cells, while those with short tails can activate and enhance the immune response to perceived threats. This classification into inhibitory and activating types underlines the dual role KIRs play in immune regulation, influencing everything from infection responses to the pathogenesis of autoimmune diseases and the success of transplanted organs [9]. Inhibitory KIRs generally promote tolerance and prevent autoimmunity by suppressing NK cell activity when they engage with compatible HLA ligands. Conversely, activating KIRs can trigger NK cell responses, leading to cell lysis if specific ligand interactions are perceived as being abnormal. This balance between activation and inhibition is crucial for maintaining self-tolerance and responding to pathological changes.

The KIR gene complex on chromosome 19q13.4 includes a variety of genes that encode both inhibitory and activating receptors. The genetic diversity of KIRs, combined with the polymorphic nature of HLA Class I molecules, creates a wide array of potential NK cell responses, influencing both the immediate and long-term outcomes of organ transplants [10].

Recent investigations have highlighted the influence of KIR and HLA compatibility on transplantation outcomes, focusing particularly on how these interactions affect NK cell reactivity, which can drastically alter the immune environment post transplantation. For instance, specific KIR-HLA interactions have been associated with either heightened risks of acute rejection or improved graft survival, underscoring the need for meticulous genetic matching in donor selection processes [11,12].

The compatibility between KIR genes and HLA types has emerged as a critical determinant in transplant immunology, influencing everything from the initial acceptance of the graft to its long-term survival. The KIR gene complex exhibits considerable polymorphism. Combination with the diverse array of HLA Class I molecules creates a myriad of potential interactions, each with unique implications for graft outcome [13]. The HLA molecules involved in these interactions are highly polymorphic, which adds another layer of complexity to the immune responses involved in transplantation [14,15]. HLA-C molecules, for instance, are classified into two groups based on their molecular structure at certain positions, which affects their interaction with specific KIRs. The presence of serine at position 77 and asparagine at position 80 defines HLA-C group 1 (C1), which interacts with KIR2DL2 and KIR2DL3, whereas lysine at position 80 defines HLA-C group 2 (C2), recognized by KIR2DL1.

In the realm of clinical transplantation, the KIR-HLA interaction impacts not only the direct cytotoxic potential of NK cells but also their role in shaping adaptive immune responses. For example, certain KIR-HLA mismatches have been correlated with an increased likelihood of developing antibody-mediated rejection, a condition where NK cells may contribute to the inflammatory milieu that harms the graft [13].

Moreover, the role of NK cells in transplant tolerance is increasingly recognized. Studies have shown that specific KIR alleles, when in the presence of compatible HLA ligands, can lead to more tolerant immunological states, potentially reducing the intensity of the immunosuppressive therapy required and improving the quality of life for transplant recipients [4,16,17,18].

Kidney transplantation is a critical procedure that requires the careful consideration of various factors, including HLA/KIR matching or mismatching. The compatibility of these factors can greatly impact the success of transplant and the long-term health of recipients. Therefore, it is vital to understand the importance of HLA/KIR matching or mismatching in kidney transplantation.

## 2. Results

The characteristics of our analyzed patients’ cohort, including those with humoral chronic rejection patients (n = 16) and those with stable renal function (n = 68), are summarized in Table 1. These characteristics include age, gender, type of donor, HLA mismatches, and Panel Reactive Antibody (PRA) level and serum creatinine levels at one year, three years, and five years post-transplantation.

Overall, 68 study participants received an organ from a deceased donor (DD) and 16 participants received an organ from a living related donor (LRD). The significantly lower median age of patients with graft rejection (29 years) compared to those without rejection (48 years) (*p* = 0.001) suggests that younger patients may have more aggressive immune responses, leading to higher rejection rates. A possible cause of this is the discontinuity of immunosuppression in young patients. The similar proportion of male patients in both groups (59.52% overall) with a *p*-value of 0.93 indicates that gender does not significantly influence graft outcomes in this study cohort.

The *p*-value of 0.412 indicates that there is no significant difference in the HLA Class I mismatch levels affecting graft outcomes. The significant *p*-value of 0.001 and mean difference of −0.59 suggest that higher levels of HLA Class II mismatches are associated with an increased risk of graft rejection. The presence of a negative sign in the mean difference indicates that the mismatch score is higher in the rejection group. The results highlight the importance of the careful consideration of recipient age and meticulous HLA matching, particularly at the HLA Class II locus, to improve transplant outcomes. Additionally, the regular monitoring of serum creatinine levels can aid in early intervention and the management of potential graft rejection.

We investigated the recipient KIR genes (inhibitory receptors 2DL1 2DL2, 2DL3, 2DL4, 2DL5, 3DL1, 3DL2, and 3DL3, and activating receptors 2DS1, 2DS3, 2DS4, 2DS5, and 3DS1).

Table 2 represents the frequencies of each KIR gene in 84 patients among recipients with and without rejection. A significant finding is the higher frequency of KIR3DL1 in patients without rejection (95.58%) compared to those with rejection (75%) (*p* = 0.007). KIR3DL1, known for its inhibitory effects, might confer protective benefits against graft rejection, potentially by moderating NK cell activity against the transplanted kidney. KIR 2DS5 (*p* = 0.001) was found to play an activating role in patients, with higher frequency among patients with CR compared to patients with SGF.

We noticed in Table 3 a notable association with an odds ratio of 6.60 and a *p*-value of 0.001 for 2DS5/HLA-C2 suggests a strong link between this activating receptor–ligand pair and graft rejection.

**Figure 1:**
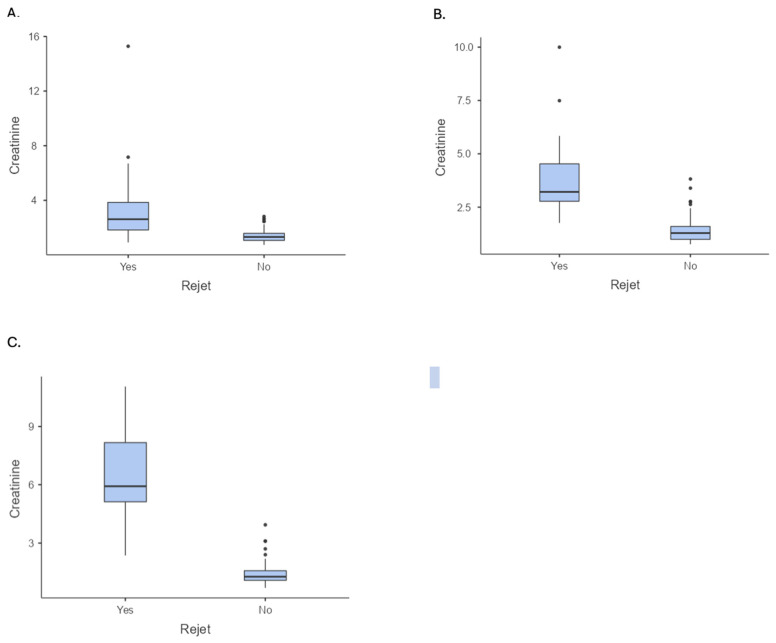
Serum creatinine levels in patients with rejection compared to those without rejection ((**A**) creatinine levels at 12 months, (**B**) creatinine levels at 36 months, and (**C**) creatinine levels at 60-month follow-up).

In this Figure 1A–C shows a dramatic increase in serum creatinine levels in patients with rejection compared to those without. This biochemical marker serves as a critical indicator of graft health.

**Figure 2:**
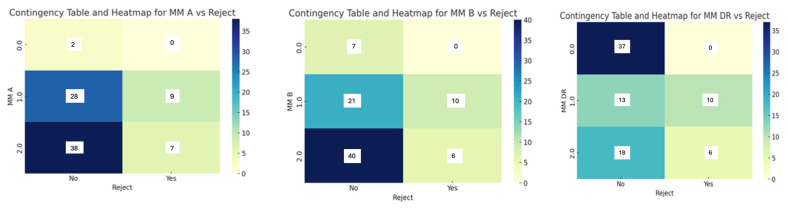
The contingency and heatmap display the relationship between mismatch loci (locus A, locus B, and DR) and graft rejection (Reject).

Understanding HLA Class I and II mismatches is critical for the pre-transplant evaluation process and long-term management of transplant recipients. The color intensity in the heatmap corresponds to the count of cases in each category, with darker shades indicating higher numbers. This visual aid helps to quickly identify which combinations of MM are most common in terms of locus A, locus B, locus DR, and rejection status (Figure 2). The heatmap illustrates that the HLA-MM at A locus is not statistically significant (*p* = 0.3674).

The absence of statistical significance suggests that the MM at A locus is not a decisive factor in renal transplantation. For the HLA-MM at B locus, this association is not statistically significant (*p* = 0.0738). A near-significant *p*-value suggests that further investigation with larger sample size could provide more insights. From 24 patients with two HLA-MM DR locus, 6 of them had rejection. These data suggest that the risk of rejection increases with the number of MM. A *p* = 0.0001 indicates a highly statistically significant relationship between HLA DR MM and graft rejection.

**Figure 3:**
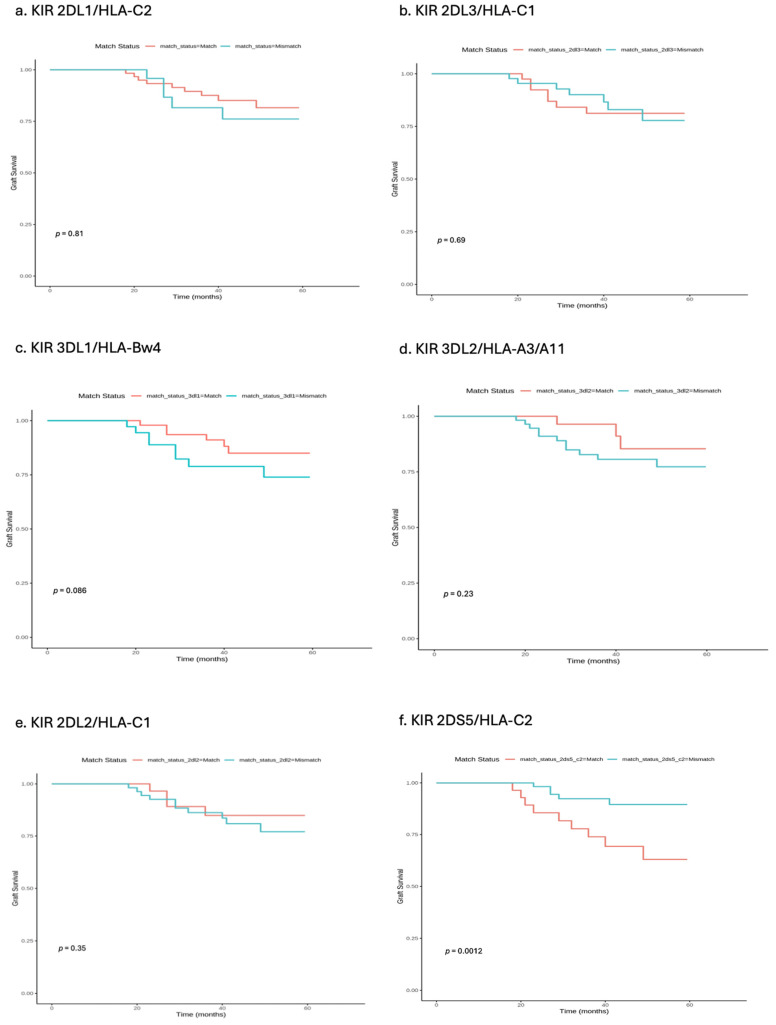

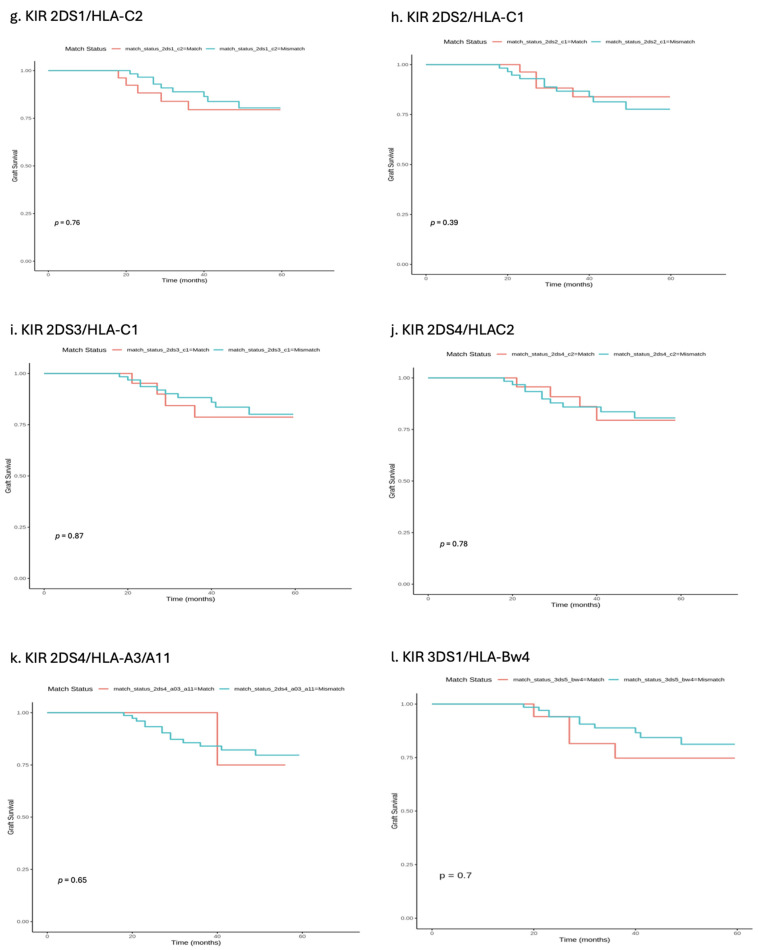
Kaplan–Meier survival curves in kidney transplant stratified by KIR/HLA ligand compatibility/incompatibility.

Figure 3 illustrates the survival probabilities up to 60 months post-transplantation, stratified by the compatibility or incompatibility of various killer immunoglobulin-like receptor (KIR) and human leukocyte antigen (HLA) ligand pairings. Each panel represents a different KIR/HLA combination, with the red line indicating compatible (match) pairings and the blue line indicating incompatible (mismatch) pairings. The *p*-values indicate the statistical significance of the differences observed between the matched and mismatched groups.

We investigated Kaplan–Meier survival curves in kidney transplants as stratified by KIR/HLA ligand compatibility/incompatibility:

1. KIR2DL1/HLA-C2: The survival curves overlap significantly, with a *p*-value of 0.81, suggesting no substantial difference in survival probabilities between compatible and incompatible pairings.
2. KIR2DL3/HLA-C1: Both groups show similar survival probabilities with a *p*-value of 0.69, indicating minimal impact of compatibility on survival outcomes.
3. KIR3DL1/HLA-Bw4: The curves are closely aligned with a *p*-value of 0.086, suggesting a slight, non-significant advantage for compatible pairings.
4. KIR3DL2/HLA-A3/A11: This graph shows a distinct separation between compatible and incompatible groups, with compatibility associated with better survival outcomes (*p*-value = 0.23).
5. KIR2DL2/HLA-C1: The survival probabilities are similar for both groups, with a *p*-value of 0.35, indicating that compatibility does not significantly affect survival.
6. KIR2DS5/HLA-C2: There is a notable divergence in survival curves after 20 months, with incompatible pairings showing decreased survival, highlighted by a significant *p*-value of 0.0012.
7. KIR2DS1/HLA-C2: There are minimal differences in survival probabilities between the groups, with a *p*-value of 0.76.
8. KIR2DS2/HLA-C1: The survival curves are closely aligned, indicating negligible impact of compatibility, with a *p*-value of 0.39.
9. KIR2DS3/HLA-C1: There are similar survival probabilities for both matches and mismatches, with a *p*-value of 0.87.
10. KIR2DS4/HLA-C2: The curves show no significant differences, with a *p*-value of 0.78.
11. KIR2DS4/HLA-A03/A11: Both groups exhibit similar survival probabilities, with a *p*-value of 0.65.
12. KIR3DS1/HLA-Bw4: The curves are closely aligned with a *p*-value of 0.7, indicating that compatibility does not significantly affect survival.

## 3. Discussion

### 3.1. KIR Genotype Frequencies and Transplant Outcomes

The data reveal the notable prevalence of KIR3DL1 among patients with stable graft function compared to those with chronic rejection, suggesting an inhibitory role that could contribute to graft tolerance. Similar findings were reported by Kunert et al. [19], who highlighted the protective role of certain KIR genes in kidney transplantation settings. The association of KIR2DS5 with poor outcomes in patients with the HLA-C2 ligand is discrepant with the findings by van Bergen et al. [20], who found that specific KIR/HLA combinations can significantly affect long-term graft survival. Although the study does not find significant differences for most KIR genes, the notable exceptions (KIR3DL1, KIR2DS5) suggest that while some KIR genes may individually contribute to transplant outcomes, the collective interaction between KIR and HLA types might be more indicative of outcome [21]. The KIR2DS5/HLA-C2 match appeared to be associated with rejection. KIR3DL1, known for its inhibitory effects, might confer protective benefits against graft rejection, potentially by moderating NK cell activity against transplanted kidneys. The lack of significant differences in most KIR genes’ frequencies suggests, that while individual KIRs may not independently predict graft outcomes, their collective balance and interaction with HLA types might be critical.

The general lack of significant differences in other KIR/HLA interactions indicates the complex nature of these relationships and underscores the necessity of considering broader immunogenetic profiles in transplant immunology [22].

### 3.2. Impact of KIR/HLA Matching

In recipients, being positive for KIR2DS5 could be indicative of an interaction where the activating receptor plays a role in dysfunctioning graft, possibly by moderating immune responses. This is in line with the study by Hanvesakul et al. [11], which demonstrated that KIR and HLA-C interactions are crucial determinants of graft failure. These findings are further supported by Yu et al. [12], who discussed the beneficial impact of matching KIR/HLA ligands in reducing acute rejection rates. The survival shown by the graphs collectively emphasizes the importance of matching KIR genes with their respective HLA ligands to improve transplant outcomes. Compatibility generally correlates with higher survival probabilities, indicating a reduced risk of graft rejection.

The degree of impact varies by KIR gene–ligand HLA pairing, with some pairings showing a more pronounced difference in outcomes between compatible and incompatible matches than others. These findings support the potential utility of considering KIR/HLA compatibility in pre-transplant immunogenetic screening in order to enhance graft survival rates and tailor immunosuppressive therapies more effectively. Tran et al. [23] and Rajalingam & Gebel [24] provide contrasting views on its impact, suggesting that in some cohorts, the mismatch might not significantly affect outcomes, pointing to the complex interplay of immune factors in transplantation. When KIR2DL1 is mismatched with HLA-C2, the survival curve indicates a more pronounced decrease in graft survival over time. The lack of inhibitory signaling due to this mismatch can lead to heightened immune activity against the graft, as outlined in studies by Rajalingam & Gebel [24], suggesting that mismatches in this critical interaction may predispose patients to increased rates of rejection.

We could see in our study that the activating receptor KIR2DS5, when mismatched with HLA-C2, also showed better survival outcomes. This was potentially due to the unregulated activation of NK cells, contributing to graft damage. The survival curves from Jafari et al. [25] underscore this risk, with incompatible pairings showing a steeper decline in survival probability. The survival curves for KIR/HLA pairings in kidney transplantation highlight the dual role of these interactions in mediating immune tolerance or activation [26,27,28,29,30]. While compatible interactions promote graft acceptance and tolerance, leading to sustained graft survival, incompatibilities increase the risk of chronic rejection. Importantly, these findings suggest that KIR/HLA matching should be considered in pre-transplant evaluations to enhance transplant outcomes. The confidence in these curves would depend significantly on the sample size and variability within the patient cohorts. Larger sample sizes would lend more robustness to wordfinding, reducing the potential errors.

## 4. Materials and Methods

This study included 84 transplant recipients who underwent kidney transplants between January 2018 and November 2020 at the Clinical Institute of Urology and Renal Transplantation Cluj-Napoca. All participants provided written consent and they had 18 years or older at the time of transplantation.

For all transplant patients, the inclusion criteria were estimated, with a glomerular filtration rate (eGFR) < 10 mL/min/1.73 m^2^. The recipients were divided into two groups: 68 patients in the stable graft function group (SGF), of which 37 patients have HLA–DRB1 identical to the HLA kidney transplant donor, and 16 patients in the chronic kidney rejection group (CR). KIR frequencies were compared among patients’ stable graft function, chronic rejection. DNA was extracted from whole blood samples using magnetic beads and innuPREP Blood DNA Mini kit IPC16 (Analytik Jena AG, Berlin, Germany). The quality of DNA was determined by a nanophotometer, and the DNA concentration was adjusted to 10–30 ng/µL.

HLA-A, -B, -C, and -DRB1 were typed via sequence-specific oligonucleotide (SSO) typing using the HISTO SPOT ABDRB1 kit (Bag Diagnostics GmbH, Lich, Germany). HLA data were analyzed with HISTO MATCH Software (V4.X-03/2020, Bag Diagnostics GmbH, Lich, Germany) according to the manufacturer’s instructions. Ambiguous HLA typing was retested via PCR-SSP using the HLA A-B-DR SSP Combi Tray (CareDx, Stockholm, Sweden) according to the manufacturer’s instructions. The results were processed with the Helmberg SCORE software version 5.00.41T.

KIR genotyping was performed using PCR-SSP to assist the presence or absence of the 16 KIR genes (KIR2DL1, KIR2DL2, KIR2DL3, KIR2DL4, KIR2DL5, KIR3DL1, KIR3DL2, KIR3DL3, KIR2DS1, KIR2DS2, KIR2DS3, KIR2DS4, KIR2DS51) [31,32]. Genotyping was performed using the commercial KIR typing kit (KIR—Ready Gene from Inno-Train Diagnostik GmbH, Kronberg, Taunus, Germany). Amplicons were visualized using 2% agarose gel electrophoresis via UV examination.

### Statistical Analysis

Descriptive statistics were performed using mean ± standard deviation for quantitative variables and median with interquartile range for qualitative variables. Inferential statistics utilized *t*-tests or Fisher’s exact tests based on distribution, with odds ratios (ORs) and 95% confidence intervals (CIs) calculated using R Commander (v. 4.2.3) and Python (v. 3.12.3). A *p*-value threshold of 0.05 was applied [33].

We conducted a survival analysis using the Kaplan–Meier model. A *p*-value < 0.05 was considered to be statistically significant [34].

## 5. Conclusions

The relationship between KIR genes and HLA ligands is a critical determinant of the success of kidney transplants, as evidenced by the survival curves. Understanding these genetic interactions offers a pathway to improving clinical protocols and patient outcomes in transplantation, emphasizing the need for continued research and innovation in this area. The survival curves extend up to 60 months, providing long-term insights into graft survival. This long-term data are essential for assessing the durability of transplant success and potential late rejection episodes. The knowledge of specific KIR/HLA interactions that influence transplant survival could guide personalized immunosuppressive therapies, possibly improving long-term outcomes.

This study opens avenues for more detailed investigations into the mechanisms by which KIR/HLA compatibilities affect immune tolerance. Further research could explore the effects of interaction with other genetic markers or non-genetic factors like patient age, comorbid conditions, and differences in immunosuppressive treatment regimens. The results of this retrospective study should be interpreted as associations between KIR and HLA that require further validation.

## Figures and Tables

**Table 1:** Patient demographics differentiating between those with stable graft function (SGF) and those with chronic rejection (CR).

	Patients	SGF	CR	*p* Value; OR ^(a)^ or x1 − x2 ^(b)^ (95% CI)
Kidney transplant patients	84	68	16	-
Patient age, years (median, IQR)	46 (32–55)	48 (37–56)	29 (23–40)	0.001; 19 ^(b)^ (−20.59 to −5.35)
Male patients, n (%)	50 (59.52)	38 (45.2)	12 (14.3)	0.93; 0.94 ^(a)^ (0.25 to 3.49)
Female patients, n (%)	34 (40.48)	30 (35.71)	4 (4.76)	0.16; 2.37 ^(a)^ (0.693 to 8.09)
DD, n (%)	68 (81.0)	56 (66.7)	12 (14.3)	0.223; 2.12 ^(a)^ (0.622 to 7.24)
LRD, n (%)	16 (19.0)	11 (13.0)	5 (6.0)	0.86; 4.25 ^(a)^ (−0.475 to 1.98)
Patient/donor HLA compatibility (mean ± SD)				
Class I (HLA-A, -B) mismatch (0–4)	2.97 ± 0.99	3.01 ± 1.02	2.81 ± 0.83	0.412; 0.2 ^(b)^ (−0.70 to 0.29)
0Class II (HLA-DR) mismatch (0–2)	0.83 ± 0.83	0.72 ± 0.86	1.31 ± 0.47	0.001; −0.59 ^(b)^ (0.27 to 0.91)
PRA, n (%)				
PRA% Class I > 50%	6 (7.14)	-	6 (7.14)	
PRA% Class II > 50%	10 (11.90)	-	10 (11.90)	
Transplantation outcome				
Serum creatinine 1 year (mean ± SD)	1.85 ± 1.83	1.39 ± 0.47	3.81 ± 3.41	0.001; −2.42 ^(b)^ (1.45 to 2.44)
Serum creatinine 3 years (mean ± SD)	1.91 ± 1.49	1.41 ± 0.59	4.03 ± 2.10	0.001; −2.62 ^(b)^ (1.58 to 2.23)
Serum creatinine 5 years (mean ± SD)	2.34 ± 2.23	1.43 ± 0.59	6.50 ± 2.16	0.001; −5.07 ^(b)^ (1.85 to 2.83)

^(a)^ Odds ratios (for categorical variables): patients with stable graft function versus patients with chronic rejection. ^(b)^ Mean differences (for continuous variables): x1 (patients with stable graft function) − x2 (patients with chronic rejection). IQR = interquartile range; CI = confidence interval; OR = odds ratio; SD = standard deviation.

**Table 2:** Frequencies of KIR genotype in chronic rejection and stable graft function.

	97 Controls n (%)	84 Patients n (%)	*p* Value	68 SGF n (%)	16 CR n (%)	*p* Value	OR (95% CI)
**Patient inhibitory KIR genes**							
2DL1	94 (96.91)	74 (89.28)	0.193	59 (86.76)	15 (93.75)	0.430	0.44 (0.05 to 3.72)
2DL2	49 (50.52)	50 (59.52)	0.268	41 (60.29)	9 (56.25)	0.766	0.85 (0.28 to 2.55)
2DL3	85 (87.63)	70 (83.33)	0.233	55 (80.88)	15 (93.75)	0.214	0.28 (0.03 to 2.33)
2DL4	72 (74.23)	64 (76.19)	0.315	51 (75)	13 (81.25)	0.597	0.69 (0.18 to 2.72)
2DL5	42 (43.30)	48 (57.14)	0.365	39 (57.35)	9 (56.25)	0.936	1.05 (0.35 to 3.14)
3DL1	84 (86.60)	77 (91.66)	0.151	65 (95.58)	12 (75)	0.007	0.14 (0.03 to 0.70)
3DL2	95 (97.94)	84 (100)	0.961	68 (100)	16 (100)	1	-
3DL3	94 (96.91)	84 (100)	0.945	68 (100)	16 (100)	1	-
**Patient activating KIR genes**							
2DS1	36 (37.11)	33 (39.28)	0.339	26 (38.23)	7 (43.75)	0.684	0.80 (0.26 to 2.40)
2DS2	50 (51.55)	41 (48.81)	0.390	33 (48.52)	8 (48.52)	0.915	0.83 (0.27 to 2.53)
2DS3	32 (32.99)	33 (39.28)	0.424	26 (38.23)	7 (43.75)	0.684	0.80 (0.26 to 2.40)
2DS4	28 (28.87)	25 (29.76)	0.466	21 (30.88)	4 (25.00)	0.643	1.34 (0.39 to 4.65)
2DS5	31 (31.96)	35 (41.66)	0.517	22 (32.35)	13 (81.25)	0.001	2.17 (0.56 to 8.41)

**Table 3:** Number of KIR–ligand HLA matches in patients with SGF and CR.

	84 Patients n (%)	68 SGF n (%)	16 CR n (%)	*p* Value	OR (95% CI)
**Recipient activating KIR genes/donor HLA ligands**					
2DS1/HLA-C2	66 (78.57)	54 (79.41)	12 (75.00)	0.698	0.78 (0.22 to 2.78)
2DS4/HLA-A*03/A*11	28 (33.33)	25 (36.76)	3 (18.75)	0.169	0.40 (0.10 to 1.53)
3DS1/HLA-Bw4	51 (60.71)	44 (64.70)	7 (43.75)	0.122	0.42 (0.14 to 1.28)
2DS2/HLA-C1	28 (33.33)	24 (35.29)	4 (25.00)	0.432	0.61 (0.17 to 2.10)
2DS3/HLA-C1	23 (33.82)	17 (25.00)	6(37.50)	0.999	1.00 (0.28 to 3.52)
2DS4/HLA-C2	21 (25.00)	19 (27.94)	2 (12.50)	0.812	0.86 (0.24 to 3.00)
2DS5/HLA-C2	28 (33.33)	17 (25.00)	9(56.25)	0.001	6.60 (2.01 to 21.7)
**Recipient inhibitory KIR genes/donor HLA ligands**					
2DL1/HLA-C2	66 (78.57)	54 (79.41)	12 (75.00)	0.698	0.78 (0.22 to 2.78)
2DL2/HLA-C1	49 (58.33)	42 (61.76)	7 (43.75)	0.188	0.48 (0.16 to 1.45)
2DL3/HLA-C1	49 (58.33)	42 (61.76)	7 (43.75)	0.188	0.48 (0.16 to 1.45)
3DL1/HLA-Bw4	51 (60.71)	44 (64.70)	7 (43.75)	0.122	0.42 (0.14 to 1.28)
3DL2/HLA-A*03/A*11	28 (33.33)	25 (36.76)	3 (18.75)	0.169	0.40 (0.10 to 1.53)

“*” represent separator between locus A and allele group.

## Data Availability

The data presented in this study are available within the article.
